# Altered Brain Activation during Emotional Face Processing in Relation to Both Diagnosis and Polygenic Risk of Bipolar Disorder

**DOI:** 10.1371/journal.pone.0134202

**Published:** 2015-07-29

**Authors:** Martin Tesli, Karolina Kauppi, Francesco Bettella, Christine Lycke Brandt, Tobias Kaufmann, Thomas Espeseth, Morten Mattingsdal, Ingrid Agartz, Ingrid Melle, Srdjan Djurovic, Lars T. Westlye, Ole A. Andreassen

**Affiliations:** 1 NORMENT, KG Jebsen Centre for Psychosis Research, Institute of Clinical Medicine, University of Oslo, Oslo, Norway; 2 Division of Mental Health and Addiction, Oslo University Hospital, Oslo, Norway; 3 Department of Psychology, University of Oslo, Oslo, Norway; 4 Sørlandet Hospital HF, Kristiansand, Norway; 5 HUBIN Project, Psychiatry Section, Department of Clinical Neuroscience, Karolinska Institutet and Hospital, Stockholm, Sweden; 6 Department of Psychiatric Research, Diakonhjemmet Hospital, Oslo, Norway; 7 Division of Medical Genetics, Oslo University Hospital, Oslo, Norway; University Medical Center Goettingen, GERMANY

## Abstract

**Objectives:**

Bipolar disorder (BD) is a highly heritable disorder with polygenic inheritance. Among the most consistent findings from functional magnetic imaging (fMRI) studies are limbic hyperactivation and dorsal hypoactivation. However, the relation between reported brain functional abnormalities and underlying genetic risk remains elusive. This is the first cross-sectional study applying a whole-brain explorative approach to investigate potential influence of BD case-control status and polygenic risk on brain activation.

**Methods:**

A BD polygenic risk score (PGRS) was estimated from the Psychiatric Genomics Consortium BD case-control study, and assigned to each individual in our independent sample (N=85 BD cases and 121 healthy controls (HC)), all of whom participated in an fMRI emotional faces matching paradigm. Potential differences in BOLD response across diagnostic groups were explored at whole-brain level in addition to amygdala as a region of interest. Putative effects of BD PGRS on brain activation were also investigated.

**Results:**

At whole-brain level, BD cases presented with significantly lower cuneus/precuneus activation than HC during negative face processing (Z-threshold=2.3 as cluster-level correction). The PGRS was associated positively with increased right inferior frontal gyrus (rIFG) activation during negative face processing. For amygdala activation, there were no correlations with diagnostic status or PGRS.

**Conclusions:**

These findings are in line with previous reports of reduced precuneus and altered rIFG activation in BD. While these results demonstrate the ability of PGRS to reveal underlying genetic risk of altered brain activation in BD, the lack of convergence of effects at diagnostic and PGRS level suggests that this relation is a complex one.

## Introduction

Bipolar disorder (BD) is a highly heritable disorder with polygenic inheritance. Large, recent studies with high statistical power have identified several genetic risk variants, most notably single nucleotide polymorphisms (SNPs) in calcium channel encoding genes [1].

In comparison, functional magnetic resonance imaging (fMRI) studies of BD have yielded inconsistent results, likely partly due to low statistical power [2]. Among the most consistently reported findings are hyperactivity of the ventral-limbic brain network and hypoactivity of dorsal brain structures [3]. A recent quantitative meta-analysis combining results from 65 fMRI studies (N = 1040 BD cases and 1074 healthy controls) found underactivation in the inferior frontal cortex and overactivation in limbic areas in BD patients relative to controls across emotional paradigms [4].

However, the relation between reported brain functional abnormalities and underlying genetic risk remains elusive. One of the most studied BD risk variants in fMRI analyses is the allele A in the *CACNA1C* SNP rs1006737, which has been related to increased amygdala activity during emotional paradigms, both in healthy controls (HC) [5], BD patients [6] and healthy relatives of BD patients [7]. Another study found carriers of the risk variant in the gene *ODZ4* (rs12576775) to be associated with increased amygdala activity in HC [8]. The genome-wide association study (GWAS) approach has also been applied to fMRI studies, of which one implicated a variant near a monoaminergic pathway gene (*PHOX2B*) in amygdala activity during a negative faces matching paradigm [9] in a sample of HC and patients.

As each susceptibility variant has been shown to have a negligible effect on the risk of BD [1], using cumulative risk load instead of single variants increases the statistical power of imaging genetics studies. A polygenic risk score (PGRS) method has been developed, which provides information on the cumulative genomic risk for BD, and accounts for a larger proportion of the phenotypic variance than single variants [10,11]). In one recent study, BD PGRS correlated positively with activation in the anterior cingulate cortex and amygdala during an executive processing/language task (N = 87 BD cases and 71 HC) across groups, with no evidence of an interaction effect between diagnostic group and PGRS on brain activation [12]. To the best of our knowledge, this is to date the only study on PGRS and fMRI in BD.

Here, we applied a whole-brain explorative approach to investigate potential differences in brain activation between BD cases (N = 85) and HC (N = 121) during an emotional faces matching fMRI paradigm. We also explored the relation between BD polygenic risk and whole-brain activity. As amygdala hyperactivity has been frequently reported in BD, we performed an additional targeted region of interest (ROI) analysis in order to investigate correlations between mean amygdala task-related activation and diagnostic status as well as BD PGRS. Additionally, differences between BD1 and BD2 were investigated, as BD1 and BD2 have been proposed to be separate disorders as well as belonging to the same BD spectrum [11].

## Materials and Methods

### Ethics Statement

The study was approved by the Regional Committee for Medical Research Ethics and the Norwegian Data Inspectorate and was conducted according to the principles of the Declaration of Helsinki. All participants were informed of the background, purpose, methods, sources of funding, potential benefits and discomforts as well as the further storage and use of the data collected in this study. Potential subjects were informed of their right to withdraw their consent at any time. Each subject provided their freely-given, written informed consent prior to the collection of data. Some patients with BD may have a reduced ability to give informed consent, but in the current study only participants with a capacity to consent were included. This was specifically assessed by the clinical recruitment teams, which included experienced clinical psychologists or psychiatrists, according to a procedure approved by the local Regional Committee for Medical Research Ethics. Potential participants who declined to participate were not disadvantaged in any way by not participating in the study, and received same quality and amount of treatment and care from the hospital as the participants.

### Sample characteristics

The total number of individuals in this study was 206, including 43 bipolar disorder type 1 (BD1) cases, 36 bipolar disorder type 2 (BD2) cases, 6 bipolar disorder not otherwise specified (BDNOS) cases, and 121 healthy controls. Our sample consisted of Northern European Caucasians, mainly Norwegians, and has previously been demonstrated to be genetically homogenous [13,14].

To be included in the study, patients had to be between 18 and 65 years, have a BD diagnosis according to the Structural Clinical Interview for DSM-IV (SCID) [15], and be willing and able to provide written informed consent. Exclusion criteria were an IQ score below 70 and reporting a history of head injury or neurological disorder. Diagnostic evaluation was performed by trained psychologists and psychiatrists, of whom all participated regularly in diagnostic meetings supervised by professors in psychiatry. Reliability measures of the diagnostic assessment in the study were performed, and the overall agreement for the DSM-IV diagnostic categories tested was 82% and the overall Kappa 0.77 (95% CI: 0.60–0.94) [16].

The healthy control subjects were recruited from the same catchment area as the patient group, were selected randomly from the national statistics records (www.ssb.no), and they all underwent an initial interview where demographic and clinical information was obtained. A history of a medical condition potentially interfering with brain function (hypothyroidism, uncontrolled hypertension and diabetes), or an illicit drug abuse/addiction diagnosis were also exclusion criteria. In the healthy control group, we also excluded subjects if they or their close relatives had a lifetime history of a severe psychiatric disorder (BD, schizophrenia and major depression).

Information on education, age of onset, number of relapses, medication status, alcohol and illegal substance abuse was obtained during an initial clinical interview. A three-hour neuropsychological test battery, including Wechsler Abbreviated Scale of Intelligence (WASI), was carried out by trained clinical psychologists.

On the day of scanning, patients underwent an abbreviated re-interview including Young Mania Rating Scale (YMRS) [17] Inventory of Depressive Symptoms (IDS) [18] and a Positive and Negative Syndrome Scale (PANSS) [19]. Information on medication status on the day of scanning was also obtained during this interview. For patients lacking data for this re-interview, we used corresponding data from the clinical interview within a time window of 3 months from the day of scanning. 26 patients presented with euthymia, 18 were mildly depressed, 10 were moderately depressed, 3 were severely depressed and 3 had symptoms of very severe depression. IDS score at scanning was available for 60/85 individuals (70.6%), for only 3 of these individuals we used information from the 3 month time window. None of the patients had elevated mood (YMRS>20) during the fMRI scanning session.

Patients and healthy controls were included from 2003 to 2009. Clinical assessment of the patients and healthy controls participating in this study is described in details in previous reports [6,20]. Demographic and clinical data are presented in Table 1.

**Table 1:** Demographic data and clinical characterization of individuals participating in a faces matching functional MRI study.

	BD (N = 85)	HC (N = 121)	P
Demographics			
Education (years), mean (SD)	13.4 (2.1)	14.2 (2.3)	0.012
N (Females, %)	52 (61.2)	53 (43.8)	0.021
Mean age (SD) a	34.8 (11.2)	35.0 (8.8)	0.88
Clinical data			
BD PGRS (SD)	0.28 (0.95)	0.2 (0.99)	0.0005
WASI, mean (SD)	110.4 (11.1)	115.3 (10.0)	0.001
IDS, mean (SD) b	17.2 (13.6)	-	-
YMRS, mean (SD) c	2.5 (3.5)	-	-
PANSS P score, mean (SD) d	10.4 (4.2)	-	-
GAF-S, mean (SD)	56.4 (11.0)	-	-
GAF-F, mean (SD)	55.1 (12.9)	-	-
Age of onset, mean (SD)	21.4 (8.3)	-	-
Duration of illness, mean (SD)	13.4 (10.1)	-	-
No. of depressive episodes, mean (SD)	8.2 (16.5)	-	-
No. of manic episodes, mean (SD)	2.4 (11.1)	-	-
No. of hypomanic episodes, mean (SD)	11.9 (34.2)	-	-
Alcohol abuseb, n (%) e	8 (9.4)	-	-
Abuse of illegal substancesb, n (%) e	7 (8.2)	-	-
Behavioral data			
Response time, negative faces [ms]	1215.0 [352.5]	1068.0 [228.9]	0.001
Response time, shapes [ms]	1012.3 [252.3]	917.1 [162.2]	0.003
Response time, positive faces [ms]	1177.7 [388.6]	1076.3 [218.7]	0.036
Accuracy rate, shapes (%)	97.0 (0.03)	97.2 (0.03)	0.44
Accuracy rate, negative faces (%)	98.4 (0.09)	99.2 (0.03)	0.44
Accuracy rate, positive faces (%)	99.2 (0.02)	99.1(0.03)	0.78
Medication	N (%)	-	-
Antipsychotics	23 (27)	-	-
Anticonvulsives	34 (40)	-	-
Antidepressants	20 (24)	-	-
Lithium	7 (8)	-	-

Abbreviations: BD, bipolar disorder; HC, healthy controls; SD, standard deviation; WASI, Wechsler Abbreviated Scale of Intelligence; IDS, Inventory of Depressive Symptoms; YMRS, Young Mania Rating Scale; PANSS P score, Positive and Negative Syndrome Scale positive subscale; GAF-S, Global Assessment of Functioning–symptom score; GAF-F, Global Assessment of Functioning–function score; BD PGRS, bipolar disorder polygenic risk score; ms, milliseconds.

BD PGRS values are reported as z-scores (with SD in brackets).

Complete behavioral data (response times and accuracy rates per condition) were available for 80/85 BD and 119/121 HC. For the remaining individuals (5 BD, 2 HC), an accuracy rate for each session (i.e. a combined rate for negative faces and shapes, and for positive faces and shapes) was available and was used to confirm that the participants paid attention to the task (accuracy rate: 97.4% and 96.0%, respectively).

^a^ Mean age at fMRI scanning. Age range was 18 to 63.

^b^ IDS score at scanning was available for 60/85 individuals (70.6%).

^c^ YMRS score at scanning was available for 69/85 individuals (81.2%).

^d^ PANSS P score at scanning was available for 38/85 individuals (44.7%).

^e^ Last six months

### Genotyping

All participants were genotyped at Expression Analysis Inc (Durham, NC, USA) using the Affymetrix Genome-Wide Human SNP Array 6.0 (Affymetrix Inc, Santa Clara, CA, USA). Quality control was performed using PLINK (version 1.07; http://pngu.mgh.harvard.edu/purcell/plink/) [21]. As a quality control, exclusions of individuals based on genotyping were made of (I) one of two duplicates, (II) one of two relatives (identity by descent (IBD) > 0.1875), (III) individuals with a recorded gender differing from that determined by X chromosome marker homozygosity, (IV) mixup-samples (calculated by pairwise genome-wide identity by state (IBS)), (V) individuals with non-European ancestry (calculated with HapMap3 and MDS) and (VI) individuals with more than 5% missing genotype data. SNPs were excluded based on (I) deviation from Hardy-Weinberg equilibrium, (II) minor allele frequency below 1% and (III) low yield (<95% in controls).

### Imputation of SNPs

Following the above mentioned quality control, the candidate SNPs were imputed with MaCH [22] (http://www.sph.umich.edu/csg/abecasis/MACH/download/1000G-PhaseI-Interim.html) using the European samples in the Phase I release of the 1000 Genomes project. SNPs not present in the 1000 Genomes reference, and SNPs with ambiguous strand alignments (A/T and G/C SNPs), were removed from the sample data sets. Imputation was a three stage process, involving (I) ChunkChromosome where the data set was broken into 2,500 SNP pieces with 500 SNP overlap (http://genome.sph.umich.edu/wiki/ChunkChromosome), (II) MaCH where each piece was phased (40 rounds, 400 states) (http://www.sph.umich.edu/csg/abecasis/MaCH/download/), and (III) Minimac where each phased piece was imputed to the 1000 Genomes European reference panel (20 rounds, 400 states) (http://genome.sph.umich.edu/wiki/Minimac). In the third stage, all imputed SNPs were provided with an estimated r^2^ score as quality metric. Exclusions were made of SNPs with an r^2^ score < 0.5, leaving 9,584,802 SNPs.

### Polygenic risk score

The BD PGRS was computed based on imputed SNPs following the method developed by Purcell et al. [10]). Using PLINK version 1.07 (http://pngu.mgh.harvard.edu/purcell/plink/) [21]), we performed a meta-analysis including all PGC substudies [1] except ours (TOP3) (N = 7278 BD cases and 8901 controls) to obtain risk allele effect sizes (ln(OR)) for all imputed SNPs. The SNPs were subsequently pruned using PLINK’s–clump option (*r* ^2^ < 0.25, 500 kb windows) to select representatives with lowest p-values from all LD blocks (209088 SNPs). A PGRS was then computed for each individual in our sample by summing up the effect sizes of the selected SNPs multiplied by the number of risk alleles expected to be carried by that individual (dosage). A total of ten PGRS were computed for BD based on different p-value thresholds (p = 1, 0.5, 0.4, 0.3, 0.2, 0.1, 0.05, 0.01, 0.001, and 0.0001) for SNP inclusion. Out of the ten PGRS we selected the one explaining most variance (Nagelkerke pseudo *r* ^2^) for further analyses. The PGRS explaining most variance is the one with p-value threshold of 0.05 (23062 SNPs). The selected BD PGRS were transformed into standard scores before proceeding with the subsequent analyses.

### Experimental paradigm

A widely used and validated faces matching paradigm was employed [9,23–25]. In this task participants select which of two stimuli (displayed at the bottom of the screen) matches a target stimulus (displayed at the top). There were two different faces matching conditions, where the images displayed were either human faces expressing anger or fear (negative faces) or faces expressing happiness (positive faces), as well as a sensorimotor control condition (shapes) in which geometrical shapes were matched in the same way. The experiment was run in two separate sessions with four blocks of either negative faces or positive faces (counterbalanced between subjects). Interleaved between these blocks, participants completed 5 blocks of the sensorimotor control task for each session. Each block consisted of 6 emotion-specific face trios derived from a standard set of facial affect pictures [26]. Each trial (faces or shapes) was presented for 5.4 seconds with no inter-stimulus interval, for a total block length of 32.6 seconds. The total paradigm lasted 310 seconds. E-prime software (version 1.0 Psychology Software Tools, Inc, Pittsburgh, PA, USA) controlled the presentations of the stimuli using VisualSystem (NordicNeuroLab, Bergen, Norway). Response times and accuracy were recorded through MR-compatible ResponseGrips (NordicNeuroLab, Bergen, Norway).

### Image acquisition

MRI data were obtained with a 1.5T Siemens Magnetom Sonata (Siemens Medical Solutions, Erlangen, Germany) supplied with a standard head coil at Oslo University Hospital. The pulse sequence used for co-registration purposes in the present context was a sagittal T1-weighted magnetization prepared rapid gradient echo (MPRAGE) with the following parameters: time of repetition (TR)/echo time (TE)/inversion time (TI) = 2730ms/3.93ms/1000ms, flip angle (FA) = 7°, field of view (FOV) = 240mm, acquisition matrix = 256x192, voxel size = 1.33x0.94x1 mm3, and 160 slices. The sequence was repeated, and the two runs were combined during post processing in order to increase signal-to-noise ratio (SNR). Patients and healthy controls were scanned consecutively.

Functional T2*-weighted images were scanned with 164 BOLD-sensitive whole brain measurements per condition, using an echo-planar imaging (EPI) pulse sequence. Each EPI volume measurement consisted of 24 axial slices with TR = 2040 ms, TE = 50 ms, FOV = 224x224 mm, FA = 90°, matrix = 64x64, a pixel size of 3 mm in the axial plane, and a slice thickness of 4 mm with 1 mm gap between slices. The first seven volumes were discarded to avoid initial steady-state effects, and the last volume was also removed, leaving 156 images for analysis.

### Image quality control, processing, and statistical analysis

All functional MRI data went through an initial quality check procedure, to detect images with poor quality due to excessive head motion, slice dropout, radiofrequency (RF) artefacts or other noise. This procedure included an investigation of the mean variance image and time-series variance plots (e.g. as suggested by the MRC CBSU, the Medical Research Council funded Cognition and Brain Sciences Unit, University of Cambridge; http://imaging.mrc-cbu.cam.ac.uk/imaging/DataDiagnostics). The variance images were examined using the nordicICE software (NordicNeuroLab, Bergen, Norway), while the time-series plots were made using the TSD*iffAna* SPM utility (developed by Matthew Brett and Volkmar Glauche; http://www.fil.ion.ucl.ac.uk/spm/ext/#TSDiffAna) in Matlab. Data was excluded if there was a systematic and specific pattern of high variance (bright areas) on the functional images as shown on the variance image, e.g. dots on axial slices or vertical lines on sagittal slices representing RF-artefacts, or horizontal lines on sagittal slices representing slice dropout. This analysis was supplemented by the TSDiffana plots, which highly confirmed the same variance patterns. In addition, all individuals with translational head motion exceeding 3 mm in either direction within a task were excluded.

The functional MRI data was preprocessed and analyzed using the fMRIB Software Library (FSL, http://www.fmrib.ox.ac.uk/fsl) [27]. Individual first-level analyses was made with the following preprocessing steps: motion correction using McFLIRT, segmentation using BET (brain extraction), spatial smoothing with a 6 mm FWHM kernel, and a high pass filter with 90 s cutoff. In the first-level analysis for each run, the onset and duration of the on blocks (positive faces and negative faces respectively) were modeled with the off blocks (geometrical shapes) as implicit baseline using the general linear model (GLM). The task design was filtered and convolved with a hemodynamic response function before the model fit. A temporal derivative was added to the model to adjust for differences in acquisition time between slices. Finally, functional images were registered to each subject’s high resolution T1 image using the FSL BBR algorithm. Thereafter, a nonlinear registration to MNI-152 standard space was made using the FSL toolbox FNIRT with 12 Degrees of freedom. Mean relative motion was estimated for each individual dataset in order to test for load and diagnosis related differences in subject motion.

### Group analyses

Whole-brain group effects were based on a random-effect model. Four contrasts were examined, Negative faces > Shapes, Positive faces > Shapes, Faces > Shapes ((Negative faces > Shapes + Positive faces > Shapes)/2) and Positive vs Negative faces. The initial cluster forming threshold was set to z = 2.3, and the resulting cluster sizes were tested using Gaussian random field theory at p < 0.05.

Differences between BD cases and HC, as well as correlations between PGRS and brain activation, were examined for all four contrasts. Additionally, potential differences between BD1 and HC, BD2 and HC, and BD1 and BD2 were explored. Age and sex were used as covariates in the main analyses of diagnosis, and in the PGRS analyses age, sex and diagnostic group was included as covariates. For both case-control and PGRS analyses, additional control analyses were made where also Wechsler Abbreviated Scale of Intelligence (WASI) and educational level were included in the model (Table 1). Findings were considered significant if they survived Bonferroni correction for 8 tests (HC vs BD and PGRS analyses for 4 contrasts). For amygdalae as ROI, average BOLD signal changes (parameter estimates) were extracted from FSL and entered into a general linear model (GLM) in the statistical software package R (http://www.r-project.org/), where sex, age, diagnostic category and PGRS where used as covariates in post hoc tests for potentially significant clusters. The amygdala ROIs were defined in accordance with the probabilistic Harvard-Oxford subcortical atlas provided with FSL, and were thresholded at 25% probability. Potential effects of medication status, dichotomized (yes/no) for antipsychotics, antidepressants, anticonvulsives and lithium, were investigated for significant clusters within BD cases. Medication status was dichotomized due to non-normal distribution of defined daily dosages as assessed by visual inspection of histograms. As some BD individuals fulfilled criteria for depression according to IDS, IDS category (0–4) was regressed against significant clusters within the cases to assess putative effect of clinical state on BOLD activation differences between BD and HC.

For demographic data, case/control comparisons of age, education, Wechsler Abbreviated Scale of Intelligence (WASI) and BD PGRS, were made using t-tests, and differences in sex frequencies was tested with the Chi^2^ test. Explained variance for BD PGRS for cases-control status was estimated with Nagelkerke pseudo R^2^. These analyses were performed in R.

## Results

### Demographics and behavioral results

BD cases and HC did not differ significantly in age, but there were more females in the BD group than among HC (P = 0.021). Patients had significantly lower education (P = 0.012) and lower cognitive function (WASI) (P = 0.001) than HC. PGRS was significantly higher in BD cases than HC (P = 0.0005) and explained 8.7% of the variance in diagnostic category status (Nagelkerke pseudo R^2^. BD patients had significantly longer response times than HC for Shapes (P = 0.003), Negative Faces (P = 0.001) and Positive Faces (P = 0.04). There were no diagnostic category differences in accuracy rates (Table 1).

### Brain activation

#### Task-related networks

Across the whole group, the Negative faces > Shapes and Positive faces > Shapes contrasts revealed BOLD signal activation in amygdala, hippocampus, occipital cortex, mid frontal gyrus, dorsolateral prefrontal cortex (dlPFC) and right precentral gyrus. A very similar activation pattern was seen in the Faces > Shapes contrast. Contrasting positive and negative faces revealed significantly increased activation in right lateral occipital cortex during negative compared to positive faces (S1 Fig).

#### Differences between bipolar disorder and healthy controls

**Fig 1:**
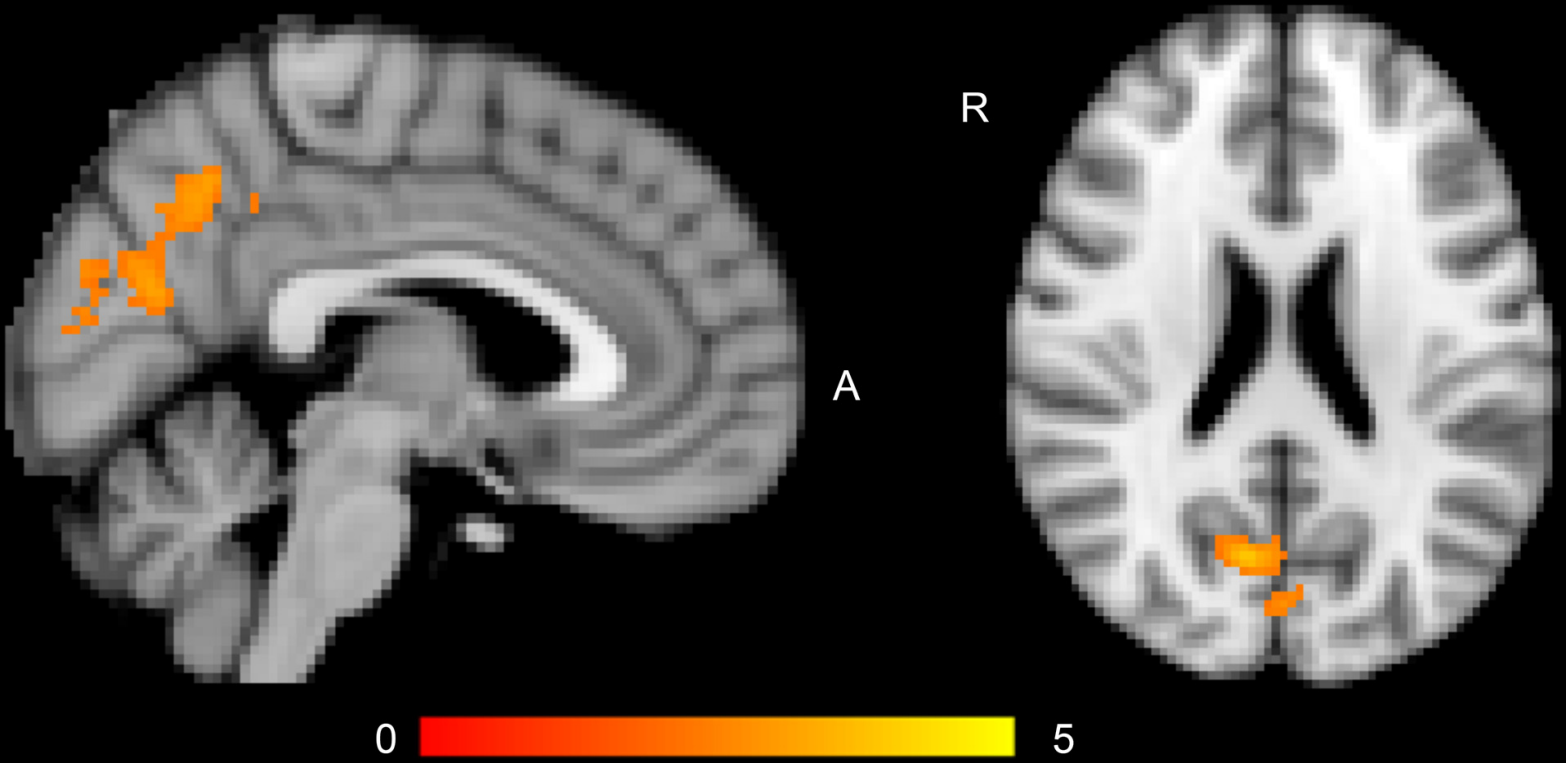
Decreased brain activation in cuneus/precuneus in bipolar disorder cases (N = 85) compared to healthy controls (N = 121) for the Negative Faces > Shapes contrast. Color bar indicates z values. Coordinates are given in MNI space. X = 4 (sagittal view), Z = 22 (transversal view). Abbreviations: R, right; A, anterior.

At whole-brain level, the main analyses of diagnosis, including age and sex as covariates, revealed lower brain activation in the cuneus/precuneus in BD patients relative to HC for the Negative faces > shapes contrast and the faces > shapes contrast (Table 2) (Fig 1). This effect remained significant only for the Negative faces > shapes contrast when including WASI (IQ) and education level as covariates, and the effect also survived Bonferroni correction for 8 test. These correlations were not significantly associated with medication status for any of the four categories (antipsychotics, antidepressants, anticonvulsives, and lithium) or with clinical state (IDS category) within the cases. For analyses of BD1 and BD2 separately, no analyses reach a Bonferroni corrected significance threshold (8 tests). At an uncorrected level, HC had increased brain activation relative to BD1 in the right prefrontal cortex for Positive faces > shapes, and in the left postcentral gyrus for Negative faces > shapes. Finally, BD2 patients had higher brain activation than HC for the Faces > shapes contrast in the lateral occipital cortex (Table 2). For ROI analyses of amygdala activity, there were no correlations with diagnostic category and no differences between BD1 and BD2 (S1 Table).

**Table 2:** Significant clusters at whole-brain level for diagnostic category and polygenic risk score analyses, corrected for sex and age.

Contrast	Group comparison	Region	Peak voxel (x y z) mm	Cluster size	Z-max	P value
**Case-control analyses**						
Neg faces > Shapes	HC > BD	Precuneus/cuneus	10, -68, 22	818	3.89	0.00012* #
Faces > Shapes	HC > BD	Cuneus	0, -80, 24	555	3.58	0.0038*
Neg faces > Shapes	HC > BD1	L Postcentral gyrus	-26, -40, 72	472	3.70	0.008
Pos faces > Shapes	HC > BD1	R prefrontal cortex	20, 70, 12	431	4.63	0.015#
Faces > Shapes	BD2 > HD	R Lateral occipital cortex	56, -70, 12	410	4.31	0.024
**PGRS analyses**						
Neg faces > Shapes	PGRS+, total sample	R Inferior frontal Gyrus	52, 18, 12	424	3.28	0.016#
Neg > Pos faces	PGRS-, total sample	R postcentral gyrus	54, -20, 50	322	3.60	0.046#

*Remains significant after Bonferroni correction (8 independent tests)

^#^P < 0.05 with IQ and education in model

Abbrevations: Pos, Positive; Neg, Negative; HC, healthy controls; BD, bipolar disorder; PGRS, polygenic risk score; L, left; R, right. ‘+’, positively associated; ‘-’, negatively associated.

Coordinates are given in MNS space.

#### Polygenic risk score

**Fig 2:**
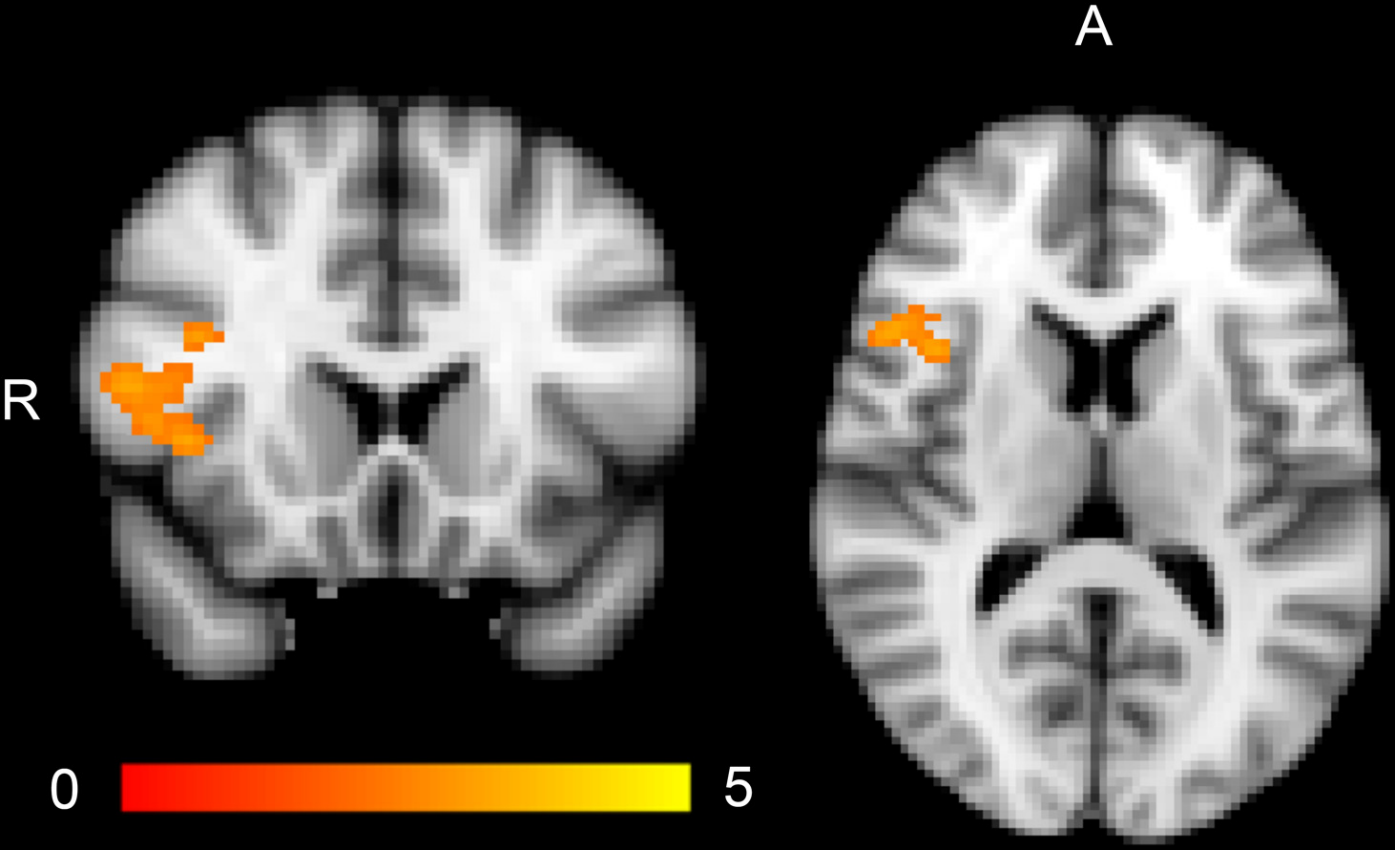
Bipolar disorder polygenic risk score is positively correlated with BOLD activation in the right inferior frontal gyrus in the entire sample (N = 206) including bipolar disorder cases and healthy controls for the Negative Faces > Shapes contrast. Color bar indicates z values. Coordinates are given in MNI space. Y = 18 (coronal view), Z = 12 (transversal view). Abbreviations: R, right; A, anterior.

PGRS was positively correlated with brain activation in the right inferior frontal gyrus (rIFG) (Table 2) (Fig 2). This effect remained significant also when WASI (IQ) and education level were used as covariates, but did not reach the Bonferroni corrected significance threshold. No significant associations between amygdala activation and PGRS were observed (S1 Table).

## Discussion

The main findings of the current study were reduced cuneus/precuneus activation in BD relative to HC, and increased rIFG activation with increasing genetic risk for BD during emotional face processing. The findings at diagnostic category level are in line with previous reports of attenuated activity in dorsal brain regions in BD, including precuneus [3]. Alterations in precuneus activity have also been reported in healthy first-degree relatives of patients with BD, as well as in paediatric BD patients [28]. These findings might indicate a genetic basis of the precuneus dysregulation in BD, although we did not find correlations between BD PGRS and precuneus activity in the current study. Another interpretation of these findings could be that dysregulations in BD patients are not due to genetics but rather secondary to the disease, and that the same pattern is seen in relatives because they manifest some of the clinical features as BD patients but to a more subtle degree. Reduced functional connectivity between precuneus and the left amygdala has also been reported in BD subjects [29], potentially pointing to alterations in the default mode network (DMN). Precuneus has been suggested to be the 'core node' or 'hub' of the DMN, a network which is thought to be activated during ‘resting consciousness’. Proposedly, this function is related to its role as a central, well connected ‘hub’ located between parietal and prefrontal regions [30]. Interestingly, hypo-connectivity was found in several DMNs with an independent component analysis (ICA) approach in BD in a large recent fMRI study [31]. In the same study, the identified connectivity patterns from these fMRI analyses were subsequently related to genes regulating specific neurodevelopment/transmission processes. As our main finding was located partly in precuneus, partly in cuneus, it is also of interest that altered activation has been reported in posterior visual and face-processing regions (i.e., right precuneus/cuneus, fusiform gyrus) in pediatric BD patients compared with HC [32].

The correlation between PGRS and increased rIFG activation (although not surviving the relatively conservative Bonferroni correction, Table 2) is in accordance with previous findings of enhanced right frontal gyrus activation in euthymic BD patients, whereas decreased activation has been reported in manic BD patients [33]. Further, hyperactivation in the IFG among BD-youths has been found to be more pronounced than in BD-adults, potentially pointing to a genetic basis [34]. At structural level, rIFG volume has been shown to be larger in BD patients than HC, but this enlargement decreased with duration of illness and was reversed with Lithium treatment [35]. IFG might be an interesting structure in BD, as it has been shown to be involved in inhibition of risk-taking behaviour [36,37], although the current findings seem rather counterintuitive in this regard, as we found enhanced IFG activation with increasing BD PGRS. However, the PGRS did not explain the identified diagnostic category differences in brain activation in the cuneus/precuneus. This indicates that underlying genetic risk for BD and diagnostic category differences in neuronal recruitment as assessed using fMRI might be of a more complex nature. Moreover, reduced cuneus/precuneus activity could be related to state processes in BD and not to underlying genetically influenced trait characteristics, as suggested in a previous report for amygdala dysregulation in schizophrenia [38]. However, level of depression was not correlated with the significant clusters distinguishing BD from HC in this sample, nor with significant clusters in the PGRS analyses.

As several studies have reported amygdala hyperactivity in BD during emotional paradigms, our lack of association suggests that this effect might have been overestimated, potentially due to small samples and the so-called ‘winner’s curse’ phenomenon [2]. Different experimental designs might also have played a role in this discrepancy, although we investigated various contrasts in this faces matching paradigm [23], as well as whole-brain activation with cluster-size correction in addition to mean amygdala activity as ROI.

With respect to the current paradigm, we applied an explorative, data-driven approach, in order to increase the possibility of identifying novel alterations in brain activation in BD, and to assess the reproducibility of previously reported patterns in a whole-brain perspective. As expected, the main effects of negative faces, positive faces, and faces in general compared to shapes across groups, all revealed strong and overlapping effects in regions comprising the amygdala, hippocampus, occipital cortex, middle frontal gyrus, dorsolateral prefrontal cortex and right precentral gyrus. Contrasting the two faces conditions, however, only revealed significant differences for negative compared to positive faces in a cluster including the right lateral occipital cortex. Thus, this paradigm seems better suited for identifying brain activation patterns during general face processing than for discriminating brain patterns during processing of faces with different emotional valence, although the task could still detect potential interaction effects between diagnostic group and activation differences between positive and negative faces. The current findings might point to dysregulated brain activation in networks underlying face processing as a characteristic of BD. This interpretation is in accordance with reports of precuneus being involved in face processing [39], as well as with impaired face recognition in BD [40]. Moreover, IFG has been implied in the face processing network [41].

There are some potential limitations to the current study, including relatively low statistical power, at least compared to recent multicenter GWAS [1]. Also, data describing emotional state at the time of scanning are lacking for some individuals (IDS data is available for 60 out of 85 BD cases (70.6%)) (Table 1), and using data from the initial clinical interview prior to scanning is an imprecise measure with the potential of giving rise to difficulties when discerning state from trait characteristics. As for the currently used PGRS, a potential limitation might be the low explained variance observed at clinical level (8.7%), leaving a large part of the genetic underpinnings of BD unaccounted for. However, this lack of explanatory power is even more problematic when using single genetic variants, with hardly observable effect sizes on clinical phenotypes [1]. Further, even though we used demographic variables as covariates, significant group differences might still influence the results.

In summary, we have found reduced relative cuneus/precuneus activation in response to emotional faces compared to shapes in BD compared with HC during a faces matching paradigm. These results are in accordance with some earlier findings, and support a model of relative hypo-activation in dorsal brain structures in BD. We also found a correlation between increased right IFG activation and increasing BD PGRS. To the best of our knowledge, this was the first study applying a whole-brain explorative approach to investigate potential influence of polygenic risk on brain activation with a face processing paradigm. However, we were not able to replicate the previously reported amygdala hyper-activation in BD. The lack of convergence of effects at case-control and PGRS level suggests a complex relation, warranting further exploration. Low explained variance for the PGRS might also limit the interpretability of the current analyses, although 8.7% is higher than the ~3% previously reported at case-control level [1,11]. Moreover, novel statistical genetic methods have already shown the potential of increasing the explained variance of cumulative genetic risk scores [42]. Future functional imaging genetics studies could also be more precise when designing fMRI paradigms, in order to disentangle trait from state characteristics, and for the purpose of combining several studies into larger, multicenter collaborative analyses.

## Supporting Information

S1 Fig Main effects of contrasts. Main effect of A. Positive Faces > Forms, B. Negative Faces > Forms and C. Negative > Positive Faces in healthy controls (N = 121) and bipolar disorder (M = 85). Color bar indicates z values. Across the whole group, the A. Positive Faces > Forms and B. Negative Faces > Forms contrasts revealed BOLD signal activation in amygdala, hippocampus, occipital cortex, mid frontal gyrus, dorsolateral prefrontal cortex and right precentral gyrus. Contrasting positive and negative faces (C) revealed significantly increased activation in right lateral occipital cortex during negative compared to positive faces. Abbreviations: Pos, Positive; Neg, Negative.
(DOCX)

S2 Fig Bar plots of diagnostic category versus parameter estimates for precuneus/cuneus activation. Bipolar disorder cases (N = 85) show decreased BOLD activation in cuneus/precuneus (x = 10, y = -68, z = 22) compared to healthy controls (N = 121) at whole-brain level (z = 2.3) for the Negative Faces > Shapes contrast. Z max = 3.89, Cluster size = 818, P = 0.00012. Parameter estimates have been selected for cluster mean value. Abbreviations: BD, bipolar disorder; NOS, not otherwise specified.
(DOCX)

S1 Table Results for case-control and polygenic risk score analyses of amygdala BOLD activation in bipolar disorder and healthy controls. Sub-tables are sorted according to contrast and laterality. Significant P values are highlighted. Abbreviations: BD, bipolar disorder; NOS, not otherwise specified; HC, healthy controls; Neg, Negative; Pos, Positive; PGRS, polygenic risk score.
(DOCX)
