# Supplementary material for: Altered Brain Activation during Emotional Face Processing in Relation to Both Diagnosis and Polygenic Risk of Bipolar Disorder
Source: PLoS One. 2015 Jul 29;10(7):e0134202. doi: 10.1371/journal.pone.0134202 (PMC4519303; doi:10.1371/journal.pone.0134202)
Supplement: S1 Fig — Main effect of A. Positive Faces > Forms, B. Negative Faces > Forms and C. Negative > Positive Faces in healthy controls (N = 121) and bipolar disorder (M = 85). Color bar indicates z values. Across the whole group, the A. Positive Faces > Forms and B. Negative Faces > Forms contrasts revealed BOLD signal activation in amygdala, hippocampus, occipital cortex, mid frontal gyrus, dorsolateral prefrontal cortex and right precentral gyrus. Contrasting positive and negative faces (C) revealed significantly increased activation in right lateral occipital cortex during negative compared to positive faces. Abbreviations: Pos, Positive; Neg, Negative. (DOCX) [file pone.0134202.s001.docx]

**
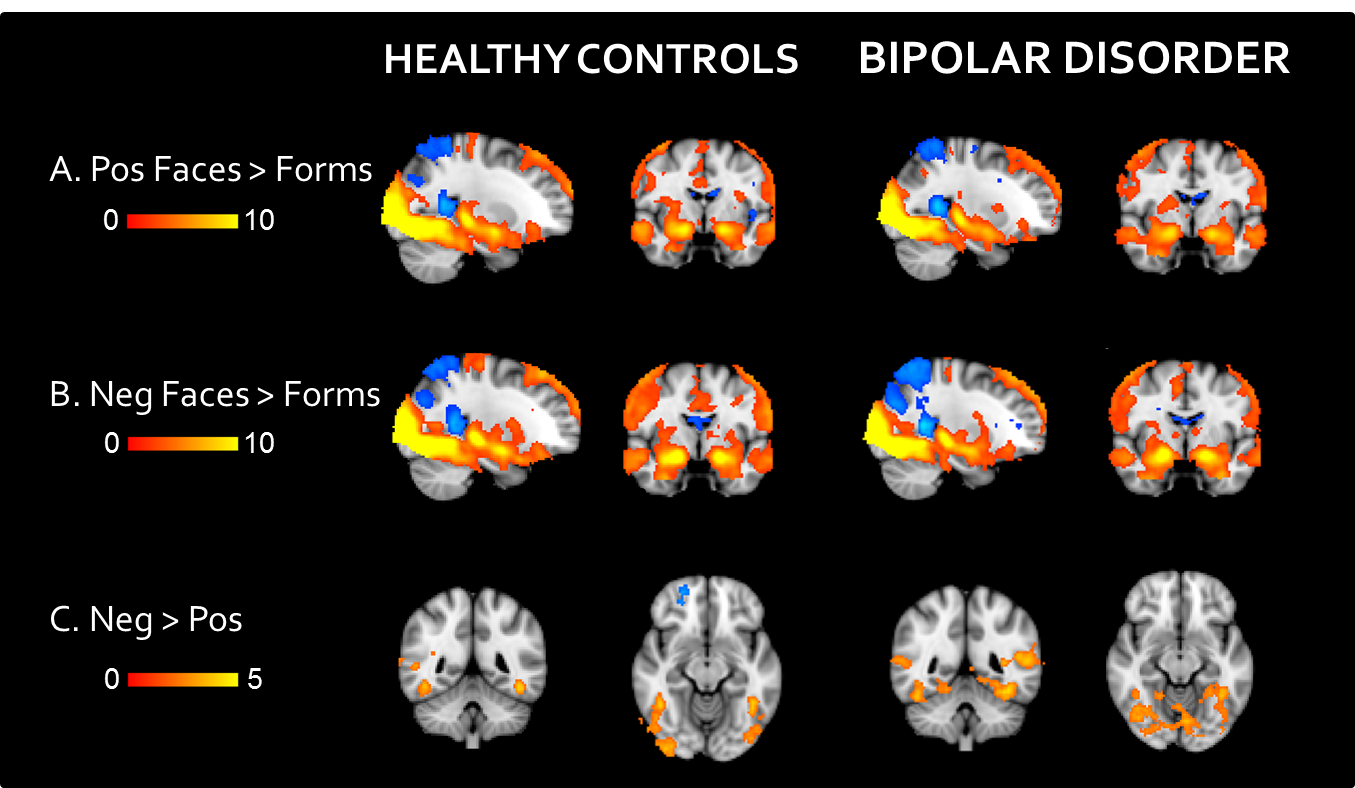
**

**S1 Fig.** Main effect of A. Positive Faces > Forms, B. Negative Faces > Forms and C. Negative > Positive Faces in healthy controls (N=121) and bipolar disorder (M=85). Color bar indicates z values. Across the whole group, the A. Positive Faces > Forms and B. Negative Faces > Forms contrasts revealed BOLD signal activation in amygdala, hippocampus, occipital cortex, mid frontal gyrus, dorsolateral prefrontal cortex and right precentral gyrus. Contrasting positive and negative faces (C) revealed significantly increased activation in right lateral occipital cortex during negative compared to positive faces.

Abbreviations: Pos, Positive; Neg, Negative.
