# Supplementary material for: Altered Brain Activation during Emotional Face Processing in Relation to Both Diagnosis and Polygenic Risk of Bipolar Disorder
Source: PLoS One. 2015 Jul 29;10(7):e0134202. doi: 10.1371/journal.pone.0134202 (PMC4519303; doi:10.1371/journal.pone.0134202)
Supplement: S2 Fig — Bipolar disorder cases (N = 85) show decreased BOLD activation in cuneus/precuneus (x = 10, y = -68, z = 22) compared to healthy controls (N = 121) at whole-brain level (z = 2.3) for the Negative Faces > Shapes contrast. Z max = 3.89, Cluster size = 818, P = 0.00012. Parameter estimates have been selected for cluster mean value. Abbreviations: BD, bipolar disorder; NOS, not otherwise specified. (DOCX) [file pone.0134202.s002.docx]

**S2 Fig. Bar plots of diagnostic category versus parameter estimates for precuneus/cuneus activation.**


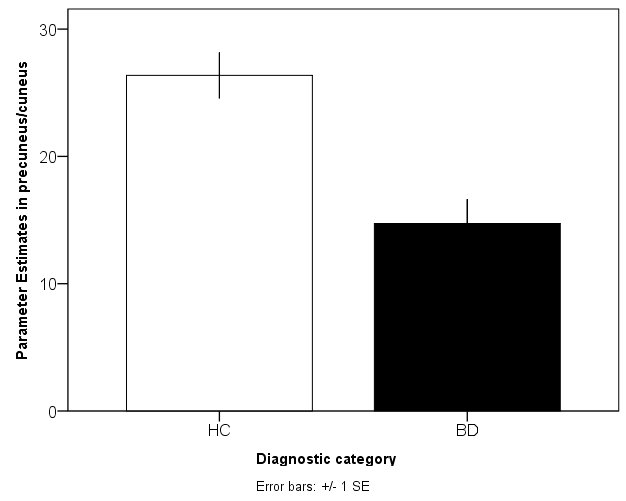


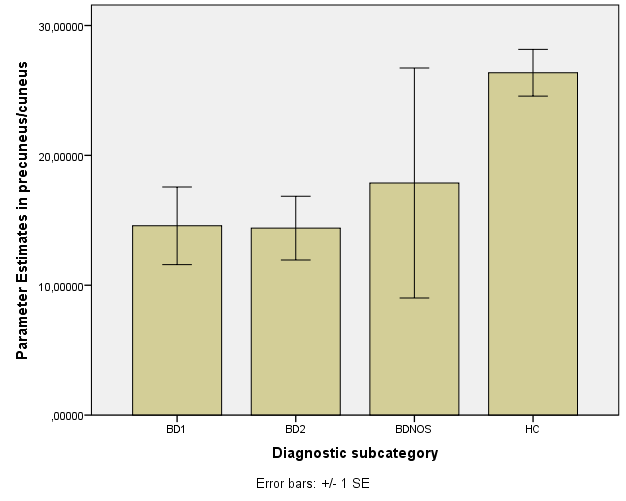


**S2 Fig.** Bipolar disorder cases (N = 85) show decreased BOLD activation in cuneus/precuneus (x = 10, y = -68, z = 22) compared to healthy controls (N = 121) at whole-brain level (z = 2.3) for the Negative Faces > Shapes contrast. Z max = 3.89, Cluster size = 818, P = 0.00012. Parameter estimates have been selected for cluster mean value.

Abbreviations: BD, bipolar disorder; NOS, not otherwise specified.
