# Supplementary material for: Altered Brain Activation during Emotional Face Processing in Relation to Both Diagnosis and Polygenic Risk of Bipolar Disorder
Source: PLoS One. 2015 Jul 29;10(7):e0134202. doi: 10.1371/journal.pone.0134202 (PMC4519303; doi:10.1371/journal.pone.0134202)
Supplement: S1 Table — Abbreviations: BD, bipolar disorder; NOS, not otherwise specified; HC, healthy controls; Neg, Negative; Pos, Positive; PGRS, polygenic risk score. (DOCX) [file pone.0134202.s003.docx]

**S1 Table. Results for case-control and polygenic risk score analyses of amygdala BOLD activation in bipolar disorder and healthy controls. Sub-tables are sorted according to contrast and laterality. Significant P values are highlighted.**

**Bipolar disorder vs healthy controls**

| Amygdala Neg Faces > Shapes left eigenvalue | | | |  |
| --- | --- | --- | --- | --- |
| Coefficients: | |  |  |  |
|  | Estimate | S.E. | t | P |
| (Intercept) | -0.03214 | 0.09483 | -0.339 | 0.735 |
| BD | 0.03979 | 0.14762 | 0.27 | 0.788 |
|  |  |  |  |  |
| Amygdala Neg Faces > Shapes left mean | | | | |
| Coefficients: | |  |  |  |
|  | Estimate | S.E. | t | P |
| (Intercept) | 13.8646 | 1.3656 | 10.153 | <2e-16 |
| BD | 0.3153 | 2.1259 | 0.148 | 0.882 |
|  |  |  |  |  |
| Amygdala Neg Faces > Shapes right eigenvalue | | | |  |
| Coefficients: | |  |  |  |
|  | Estimate | S.E. | t | P |
| (Intercept) | -0.0504 | 0.094 | -0.536 | 0.592 |
| BD | 0.0438 | 0.1463 | 0.299 | 0.765 |
|  |  |  |  |  |
| Amygdala Neg Faces > Shapes right mean | | | | |
| Coefficients: | |  |  |  |
|  | Estimate | S.E. | t | P |
| (Intercept) | 14.7045 | 1.3875 | 10.6 | <2e-16 |
| BD | 0.4755 | 2.16 | 0.22 | 0.826 |
|  |  |  |  |  |
| Amygdala Pos Faces > Shapes left eigenvalue | | | |  |
| Coefficients: | |  |  |  |
|  | Estimate | S.E. | t | P |
| (Intercept) | 0.06727 | 0.09376 | 0.717 | 0.474 |
| BD | -0.17299 | 0.14597 | -1.185 | 0.237 |
|  |  |  |  |  |
| Amygdala Pos Faces > Shapes left mean | | | | |
| Coefficients: | |  |  |  |
|  | Estimate | S.E. | t | P |
| (Intercept) | 12.341 | 1.303 | 9.468 | <2e-16 |
| BD | -2.215 | 2.029 | -1.091 | 0.276 |
|  |  |  |  |  |
| Amygdala Pos Faces > Shapes right eigenvalue | | | |  |
| Coefficients: | |  |  |  |
|  | Estimate | S.E. | t | P |
| (Intercept) | 0.1322 | 0.0922 | 1.434 | 0.1531 |
| BD | -0.2668 | 0.1435 | -1.859 | 0.0645 |
|  |  |  |  |  |
| Amygdala Pos Faces > Shapes right mean | | | | |
| Coefficients: | |  |  |  |
|  | Estimate | S.E. | t | P |
| (Intercept) | 13.397 | 1.225 | 10.934 | <2e-16 |
| BD | -3.024 | 1.907 | -1.586 | 0.114 |
|  |  |  |  |  |
| Amygdala Faces > Shapes left eigenvalue | | | |  |
| Coefficients: | |  |  |  |
|  | Estimate | S.E. | t | P |
| (Intercept) | 0.01987 | 0.09448 | 0.21 | 0.834 |
| BD | -0.08721 | 0.14709 | -0.593 | 0.554 |
|  |  |  |  |  |
| Amygdala Faces > Shapes left mean | | | |  |
| Coefficients: | |  |  |  |
|  | Estimate | S.E. | t | P |
| (Intercept) | 26.206 | 2.103 | 12.46 | <2e-16 |
| BD | -1.899 | 3.274 | -0.58 | 0.562 |
|  |  |  |  |  |
| Amygdala Faces > Shapes right eigenvalue | | | |  |
| Coefficients: | |  |  |  |
|  | Estimate | S.E. | t | P |
| (Intercept) | 0.04727 | 0.093 | 0.508 | 0.612 |
| BD | -0.13156 | 0.14478 | -0.909 | 0.365 |
|  |  |  |  |  |
| Amygdala Faces > Shapes right mean | | | |  |
| Coefficients: | |  |  |  |
|  | Estimate | S.E. | t | P |
| (Intercept) | 28.101 | 2.099 | 13.39 | <2e-16 |
| BD | -2.549 | 3.267 | -0.78 | 0.436 |
|  |  |  |  |  |
| Amygdala Neg > Pos Faces left eigenvalue | | | |  |
| Coefficients: | |  |  |  |
|  | Estimate | S.E. | t | P |
| (Intercept) | -0.08055 | 0.09356 | -0.861 | 0.39 |
| BD | 0.17502 | 0.14565 | 1.202 | 0.231 |
|  |  |  |  |  |
| Amygdala Neg > Pos Faces left mean | | | |  |
| Coefficients: | |  |  |  |
|  | Estimate | S.E. | t | P |
| (Intercept) | 1.523 | 1.645 | 0.926 | 0.356 |
| BD | 2.53 | 2.561 | 0.988 | 0.324 |
|  |  |  |  |  |
| Amygdala Neg > Pos Faces right eigenvalue | | | |  |
| Coefficients: | |  |  |  |
|  | Estimate | S.E. | t | P |
| (Intercept) | -0.14758 | 0.09306 | -1.586 | 0.1143 |
| BD | 0.25206 | 0.14487 | 1.74 | 0.0834 |
|  |  |  |  |  |
| Amygdala Neg > Pos Faces right mean | | | |  |
| Coefficients: | |  |  |  |
|  | Estimate | S.E. | t | P |
| (Intercept) | 1.308 | 1.565 | 0.836 | 0.404 |
| BD | 3.5 | 2.436 | 1.437 | 0.152 |

**Bipolar type 1 disorder vs healthy controls**

| Amygdala Neg Faces > Shapes left eigenvalue | | | |  |
| --- | --- | --- | --- | --- |
| Coefficients: | |  |  |  |
|  | Estimate | S.E. | t | P |
| (Intercept) | -0.03214 | 0.09151 | -0.351 | 0.726 |
| BD1 | -0.02773 | 0.17871 | -0.155 | 0.877 |
|  |  |  |  |  |
| Amygdala Neg Faces > Shapes left mean | | | |  |
| Coefficients: | |  |  |  |
|  | Estimate | S.E. | t | P |
| (Intercept) | 13.86459 | 1.30186 | 10.65 | <2e-16 |
| BD1 | 0.09434 | 2.54245 | 0.037 | 0.97 |
|  |  |  |  |  |
| Amygdala Neg Faces > Shapes right eigenvalue | | | |  |
| Coefficients: | |  |  |  |
|  | Estimate | S.E. | t | P |
| (Intercept) | -0.0504 | 0.08514 | -0.592 | 0.555 |
| BD1 | -0.04108 | 0.16628 | -0.247 | 0.805 |
|  |  |  |  |  |
| Amygdala Neg Faces > Shapes right mean | | | | |
| Coefficients: | |  |  |  |
|  | Estimate | S.E. | t | P |
| (Intercept) | 14.704 | 1.248 | 11.78 | <2e-16 |
| BD1 | -0.282 | 2.438 | -0.116 | 0.908 |
|  |  |  |  |  |
| Amygdala Pos Faces > Shapes left eigenvalue | | | |  |
| Coefficients: | |  |  |  |
|  | Estimate | S.E. | t | P |
| (Intercept) | 0.06727 | 0.08557 | 0.786 | 0.433 |
| BD1 | -0.15831 | 0.16712 | -0.947 | 0.345 |
|  |  |  |  |  |
| Amygdala Pos Faces > Shapes left mean | | | |  |
| Coefficients: | |  |  |  |
|  | Estimate | S.E. | t | P |
| (Intercept) | 12.341 | 1.23 | 10.036 | <2e-16 |
| BD1 | -1.883 | 2.401 | -0.784 | 0.434 |
|  |  |  |  |  |
| Amygdala Pos Faces > Shapes right eigenvalue | | | |  |
| Coefficients: | |  |  |  |
|  | Estimate | S.E. | t | P |
| (Intercept) | 0.13221 | 0.09093 | 1.454 | 0.148 |
| BD1 | -0.25625 | 0.17757 | -1.443 | 0.151 |
|  |  |  |  |  |
| Amygdala Pos Faces > Shapes right mean | | | | |
| Coefficients: | |  |  |  |
|  | Estimate | S.E. | t | P |
| (Intercept) | 13.397 | 1.217 | 11.006 | <2e-16 |
| BD1 | -2.219 | 2.377 | -0.934 | 0.352 |
|  |  |  |  |  |
| Amygdala Faces > Shapes left eigenvalue | | | |  |
| Coefficients: | |  |  |  |
|  | Estimate | S.E. | t | P |
| (Intercept) | 0.01987 | 0.09014 | 0.22 | 0.826 |
| BD1 | -0.11554 | 0.17604 | -0.656 | 0.513 |
|  |  |  |  |  |
| Amygdala Faces > Shapes left mean | | | |  |
| Coefficients: | |  |  |  |
|  | Estimate | S.E. | t | P |
| (Intercept) | 26.206 | 2.017 | 12.993 | <2e-16 |
| BD1 | -1.789 | 3.939 | -0.454 | 0.65 |
|  |  |  |  |  |
| Amygdala Faces > Shapes right eigenvalue | | | |  |
| Coefficients: | |  |  |  |
|  | Estimate | S.E. | t | P |
| (Intercept) | 0.04727 | 0.08517 | 0.555 | 0.58 |
| BD1 | -0.17689 | 0.16633 | -1.063 | 0.289 |
|  |  |  |  |  |
| Amygdala Faces > Shapes right mean | | | |  |
| Coefficients: | |  |  |  |
|  | Estimate | S.E. | t | P |
| (Intercept) | 28.101 | 1.917 | 14.659 | <2e-16 |
| BD1 | -2.501 | 3.744 | -0.668 | 0.505 |
|  |  |  |  |  |
| Amygdala Neg > Pos Faces left eigenvalue | | | |  |
| Coefficients: | |  |  |  |
|  | Estimate | S.E. | t | P |
| (Intercept) | -0.08055 | 0.08686 | -0.927 | 0.355 |
| BD1 | 0.09099 | 0.16963 | 0.536 | 0.592 |
|  |  |  |  |  |
| Amygdala Neg > Pos Faces left mean | | | |  |
| Coefficients: | |  |  |  |
|  | Estimate | S.E. | t | P |
| (Intercept) | 1.523 | 1.532 | 0.995 | 0.321 |
| BD1 | 1.977 | 2.991 | 0.661 | 0.51 |
|  |  |  |  |  |
| Amygdala Neg > Pos Faces right eigenvalue | | | |  |
| Coefficients: | |  |  |  |
|  | Estimate | S.E. | t | P |
| (Intercept) | -0.14758 | 0.09297 | -1.587 | 0.114 |
| BD1 | 0.15125 | 0.18156 | 0.833 | 0.406 |
|  |  |  |  |  |
| Amygdala Neg > Pos Faces right mean | | | |  |
| Coefficients: | |  |  |  |
|  | Estimate | S.E. | t | P |
| (Intercept) | 1.308 | 1.551 | 0.843 | 0.4 |
| BD1 | 1.937 | 3.028 | 0.64 | 0.523 |

**Bipolar type 2 disorder vs healthy controls**

| Amygdala Neg Faces > Shapes left eigenvalue | | | |  |
| --- | --- | --- | --- | --- |
| Coefficients: | |  |  |  |
|  | Estimate | S.E. | t | P |
| (Intercept) | -0.03214 | 0.09002 | -0.357 | 0.722 |
| BD2 | 0.01293 | 0.18799 | 0.069 | 0.945 |
|  |  |  |  |  |
| Amygdala Neg Faces > Shapes left mean | | | |  |
| Coefficients: | |  |  |  |
|  | Estimate | S.E. | t | P |
| (Intercept) | 13.8646 | 1.282 | 10.815 | <2e-16 |
| BD2 | -0.9841 | 2.6773 | -0.368 | 0.714 |
|  |  |  |  |  |
| Amygdala Neg Faces > Shapes right eigenvalue | | | |  |
| Coefficients: | |  |  |  |
|  | Estimate | S.E. | t | P |
| (Intercept) | -0.0504 | 0.089976 | -0.56 | 0.576 |
| BD2 | 0.009887 | 0.1879 | 0.053 | 0.958 |
|  |  |  |  |  |
| Amygdala Neg Faces > Shapes right mean | | | | |
| Coefficients: | |  |  |  |
|  | Estimate | S.E. | t | P |
| (Intercept) | 14.7045 | 1.3459 | 10.926 | <2e-16 |
| BD2 | -0.4913 | 2.8106 | -0.175 | 0.861 |
|  |  |  |  |  |
| Amygdala Pos Faces > Shapes left eigenvalue | | | |  |
| Coefficients: | |  |  |  |
|  | Estimate | S.E. | t | P |
| (Intercept) | 0.06727 | 0.09493 | 0.709 | 0.48 |
| BD2 | -0.23637 | 0.19825 | -1.192 | 0.235 |
|  |  |  |  |  |
| Amygdala Pos Faces > Shapes left mean | | | |  |
| Coefficients: | |  |  |  |
|  | Estimate | S.E. | t | P |
| (Intercept) | 12.341 | 1.326 | 9.31 | <2e-16 |
| BD2 | -3.236 | 2.768 | -1.169 | 0.244 |
|  |  |  |  |  |
| Amygdala Pos Faces > Shapes right eigenvalue | | | |  |
| Coefficients: | |  |  |  |
|  | Estimate | S.E. | t | P |
| (Intercept) | 0.13221 | 0.09115 | 1.45 | 0.1489 |
| BD2 | -0.31546 | 0.19035 | -1.657 | 0.0995 |
|  |  |  |  |  |
| Amygdala Pos Faces > Shapes right mean | | | |  |
| Coefficients: | |  |  |  |
|  | Estimate | S.E. | t | P |
| (Intercept) | 13.397 | 1.218 | 10.997 | <2e-16 |
| BD2 | -4.319 | 2.544 | -1.698 | 0.0916 |
|  |  |  |  |  |
| Amygdala Faces > Shapes left eigenvalue | | | |  |
| Coefficients: | |  |  |  |
|  | Estimate | S.E. | t | P |
| (Intercept) | 0.01987 | 0.09364 | 0.212 | 0.832 |
| BD2 | -0.15156 | 0.19555 | -0.775 | 0.439 |
|  |  |  |  |  |
| Amygdala Faces > Shapes left mean | | | |  |
| Coefficients: | |  |  |  |
|  | Estimate | S.E. | t | P |
| (Intercept) | 26.206 | 2.069 | 12.664 | <2e-16 |
| BD2 | -4.22 | 4.321 | -0.977 | 0.33 |
|  |  |  |  |  |
| Amygdala Faces > Shapes right eigenvalue | | | |  |
| Coefficients: | |  |  |  |
|  | Estimate | S.E. | t | P |
| (Intercept) | 0.04727 | 0.08847 | 0.534 | 0.594 |
| BD2 | -0.18522 | 0.18475 | -1.003 | 0.318 |
|  |  |  |  |  |
| Amygdala Faces > Shapes right mean | | | |  |
| Coefficients: | |  |  |  |
|  | Estimate | S.E. | t | P |
| (Intercept) | 28.101 | 2.024 | 13.882 | <2e-16 |
| BD2 | -4.81 | 4.227 | -1.138 | 0.257 |
|  |  |  |  |  |
| Amygdala Neg > Pos Faces left eigenvalue | | | |  |
| Coefficients: | |  |  |  |
|  | Estimate | S.E. | t | P |
| (Intercept) | -0.08055 | 0.09085 | -0.887 | 0.377 |
| BD2 | 0.23141 | 0.18973 | 1.22 | 0.224 |
|  |  |  |  |  |
| Amygdala Neg > Pos Faces left mean | | | |  |
| Coefficients: | |  |  |  |
|  | Estimate | S.E. | t | P |
| (Intercept) | 1.523 | 1.587 | 0.96 | 0.339 |
| BD2 | 2.252 | 3.315 | 0.679 | 0.498 |
|  |  |  |  |  |
| Amygdala Neg > Pos Faces right eigenvalue | | | |  |
| Coefficients: | |  |  |  |
|  | Estimate | S.E. | t | P |
| (Intercept) | -0.14758 | 0.09357 | -1.577 | 0.117 |
| BD2 | 0.28482 | 0.1954 | 1.458 | 0.147 |
|  |  |  |  |  |
| Amygdala Neg > Pos Faces right mean | | | |  |
| Coefficients: | |  |  |  |
|  | Estimate | S.E. | t | P |
| (Intercept) | 1.308 | 1.579 | 0.828 | 0.409 |
| BD2 | 3.828 | 3.297 | 1.161 | 0.247 |

**Bipolar type 1 disorder vs Bipolar type 2 disorder**

| Amygdala Neg Faces > Shapes left eigenvalue | | | |  |
| --- | --- | --- | --- | --- |
| Coefficients: | |  |  |  |
|  | Estimate | S.E. | t | P |
| (Intercept) | -0.01921 | 0.16556 | -0.116 | 0.908 |
| BD1 | -0.04066 | 0.22441 | -0.181 | 0.857 |
|  |  |  |  |  |
| Amygdala Neg Faces > Shapes left mean | | | |  |
| Coefficients: | |  |  |  |
|  | Estimate | S.E. | t | P |
| (Intercept) | 12.88 | 2.386 | 5.398 | 7.23E-07 |
| BD1 | 1.078 | 3.235 | 0.333 | 0.74 |
|  |  |  |  |  |
| Amygdala Neg Faces > Shapes right eigenvalue | | | |  |
| Coefficients: | |  |  |  |
|  | Estimate | S.E. | t | P |
| (Intercept) | -0.04052 | 0.16561 | -0.245 | 0.807 |
| BD1 | -0.05097 | 0.22448 | -0.227 | 0.821 |
|  |  |  |  |  |
| Amygdala Neg Faces > Shapes right mean | | | | |
| Coefficients: | |  |  |  |
|  | Estimate | S.E. | t | P |
| (Intercept) | 14.2132 | 2.4949 | 5.697 | 2.13E-07 |
| BD1 | 0.2093 | 3.3817 | 0.062 | 0.951 |
|  |  |  |  |  |
| Amygdala Pos Faces > Shapes left eigenvalue | | | |  |
| Coefficients: | |  |  |  |
|  | Estimate | S.E. | t | P |
| (Intercept) | -0.16911 | 0.19229 | -0.879 | 0.382 |
| BD1 | 0.07806 | 0.26064 | 0.299 | 0.765 |
|  |  |  |  |  |
| Amygdala Pos Faces > Shapes left mean | | | |  |
| Coefficients: | |  |  |  |
|  | Estimate | S.E. | t | P |
| (Intercept) | 9.105 | 2.515 | 3.621 | 0.000524 |
| BD1 | 1.353 | 3.409 | 0.397 | 0.692429 |
|  |  |  |  |  |
| Amygdala Pos Faces > Shapes right eigenvalue | | | |  |
| Coefficients: | |  |  |  |
|  | Estimate | S.E. | t | P |
| (Intercept) | -0.18325 | 0.18009 | -1.018 | 0.312 |
| BD1 | 0.05921 | 0.24409 | 0.243 | 0.809 |
|  |  |  |  |  |
| Amygdala Pos Faces > Shapes right mean | | | | |
| Coefficients: | |  |  |  |
|  | Estimate | S.E. | t | P |
| (Intercept) | 9.078 | 2.328 | 3.9 | 0.000204 |
| BD1 | 2.1 | 3.155 | 0.666 | 0.507668 |
|  |  |  |  |  |
| Amygdala Faces > Shapes left eigenvalue | | | |  |
| Coefficients: | |  |  |  |
|  | Estimate | S.E. | t | P |
| (Intercept) | -0.13169 | 0.16505 | -0.798 | 0.427 |
| BD1 | 0.03602 | 0.22371 | 0.161 | 0.873 |
|  |  |  |  |  |
| Amygdala Faces > Shapes left mean | | | |  |
| Coefficients: | |  |  |  |
|  | Estimate | S.E. | t | P |
| (Intercept) | 21.986 | 3.637 | 6.045 | 5.00E-08 |
| BD1 | 2.432 | 4.93 | 0.493 | 0.623 |
|  |  |  |  |  |
| Amygdala Faces > Shapes right eigenvalue | | | |  |
| Coefficients: | |  |  |  |
|  | Estimate | S.E. | t | P |
| (Intercept) | -0.13796 | 0.182429 | -0.756 | 0.452 |
| BD1 | 0.008334 | 0.24727 | 0.034 | 0.973 |
|  |  |  |  |  |
| Amygdala Faces > Shapes right mean | | | |  |
| Coefficients: | |  |  |  |
|  | Estimate | S.E. | t | P |
| (Intercept) | 23.291 | 4.099 | 5.683 | 2.26E-07 |
| BD1 | 2.309 | 5.556 | 0.416 | 0.679 |
|  |  |  |  |  |
| Amygdala Neg > Pos Faces left eigenvalue | | | |  |
| Coefficients: | |  |  |  |
|  | Estimate | S.E. | t | P |
| (Intercept) | 0.1509 | 0.196 | 0.77 | 0.444 |
| BD1 | -0.1404 | 0.2657 | -0.529 | 0.599 |
|  |  |  |  |  |
| Amygdala Neg > Pos Faces left mean | | | |  |
| Coefficients: | |  |  |  |
|  | Estimate | S.E. | t | P |
| (Intercept) | 3.7753 | 3.2877 | 1.148 | 0.254 |
| BD1 | -0.2749 | 4.4563 | -0.062 | 0.951 |
|  |  |  |  |  |
| Amygdala Neg > Pos Faces right eigenvalue | | | |  |
| Coefficients: | |  |  |  |
|  | Estimate | S.E. | t | P |
| (Intercept) | 0.1372 | 0.1496 | 0.917 | 0.362 |
| BD1 | -0.1336 | 0.2028 | -0.659 | 0.512 |
|  |  |  |  |  |
| Amygdala Neg > Pos Faces right mean | | | |  |
| Coefficients: | |  |  |  |
|  | Estimate | S.E. | t | P |
| (Intercept) | 5.135 | 2.546 | 2.017 | 0.0472 |
| BD1 | -1.891 | 3.452 | -0.548 | 0.5855 |

**Polygenic risk score vs amygdala activation in total sample (N=206)**

| Amygdala Neg Faces > Shapes left eigenvalue | | | |  |
| --- | --- | --- | --- | --- |
| Coefficients: | |  |  |  |
|  | Estimate | S.E. | t | P |
| (Intercept) | -0.01572 | 0.07255 | -0.217 | 0.829 |
| PRGS | -0.06503 | 0.07272 | -0.894 | 0.372 |
|  |  |  |  |  |
| Amygdala Neg Faces > Shapes left mean | | | |  |
| Coefficients: | |  |  |  |
|  | Estimate | S.E. | t | P |
| (Intercept) | 13.995 | 1.044 | 13.409 | <2e-16 |
| PRGS | -1.129 | 1.046 | -1.079 | 0.282 |
|  |  |  |  |  |
| Amygdala Neg Faces > Shapes right eigenvalue | | | |  |
| Coefficients: | |  |  |  |
|  | Estimate | S.E. | t | P |
| (Intercept) | -0.03233 | 0.07184 | -0.45 | 0.653 |
| PRGS | -0.07912 | 0.07202 | -1.099 | 0.273 |
|  |  |  |  |  |
| Amygdala Neg Faces > Shapes right mean | | | | |
| Coefficients: | |  |  |  |
|  | Estimate | S.E. | t | P |
| (Intercept) | 14.901 | 1.06 | 14.06 | <2e-16 |
| PRGS | -1.266 | 1.062 | -1.191 | 0.235 |
|  |  |  |  |  |
| Amygdala Pos Faces > Shapes left eigenvalue | | | |  |
| Coefficients: | |  |  |  |
|  | Estimate | S.E. | t | P |
| (Intercept) | -0.00411 | 0.072065 | -0.057 | 0.955 |
| PRGS | 0.035973 | 0.072241 | 0.498 | 0.619 |
|  |  |  |  |  |
| Amygdala Pos Faces > Shapes left mean | | | |  |
| Coefficients: | |  |  |  |
|  | Estimate | S.E. | t | P |
| (Intercept) | 11.4275 | 1.0007 | 11.42 | <2e-16 |
| PRGS | 0.7109 | 1.0031 | 0.709 | 0.479 |
|  |  |  |  |  |
| Amygdala Pos Faces > Shapes right eigenvalue | | | |  |
| Coefficients: | |  |  |  |
|  | Estimate | S.E. | t | P |
| (Intercept) | 0.02212 | 0.07124 | 0.31 | 0.757 |
| PRGS | 0.02565 | 0.07141 | 0.359 | 0.72 |
|  |  |  |  |  |
| Amygdala Pos Faces > Shapes right mean | | | |  |
| Coefficients: | |  |  |  |
|  | Estimate | S.E. | t | P |
| (Intercept) | 12.149 | 0.9446 | 12.862 | <2e-16 |
| PRGS | 0.3135 | 0.9469 | 0.331 | 0.741 |
|  |  |  |  |  |
| Amygdala Faces > Shapes left eigenvalue | | | |  |
| Coefficients: | |  |  |  |
|  | Estimate | S.E. | t | P |
| (Intercept) | -0.01611 | 0.07246 | -0.222 | 0.824 |
| PRGS | -0.01912 | 0.07264 | -0.263 | 0.793 |
|  |  |  |  |  |
| Amygdala Faces > Shapes left mean | | | |  |
| Coefficients: | |  |  |  |
|  | Estimate | S.E. | t | P |
| (Intercept) | 25.4222 | 1.6127 | 15.764 | <2e-16 |
| PRGS | -0.4182 | 1.6166 | -0.259 | 0.796 |
|  |  |  |  |  |
| Amygdala Faces > Shapes right eigenvalue | | | |  |
| Coefficients: | |  |  |  |
|  | Estimate | S.E. | t | P |
| (Intercept) | -0.00702 | 0.071382 | -0.098 | 0.922 |
| PRGS | -0.03344 | 0.071555 | -0.467 | 0.641 |
|  |  |  |  |  |
| Amygdala Faces > Shapes right mean | | | |  |
| Coefficients: | |  |  |  |
|  | Estimate | S.E. | t | P |
| (Intercept) | 27.05 | 1.609 | 16.81 | <2e-16 |
| PRGS | -0.952 | 1.613 | -0.59 | 0.556 |
|  |  |  |  |  |
| Amygdala Neg > Pos Faces left eigenvalue | | | |  |
| Coefficients: | |  |  |  |
|  | Estimate | S.E. | t | P |
| (Intercept) | -0.00834 | 0.071804 | -0.116 | 0.908 |
| PRGS | -0.06737 | 0.071979 | -0.936 | 0.35 |
|  |  |  |  |  |
| Amygdala Neg > Pos Faces left mean | | | |  |
| Coefficients: | |  |  |  |
|  | Estimate | S.E. | t | P |
| (Intercept) | 2.567 | 1.257 | 2.042 | 0.0424 |
| PRGS | -1.84 | 1.26 | -1.46 | 0.1458 |
|  |  |  |  |  |
| Amygdala Neg > Pos Faces right eigenvalue | | | |  |
| Coefficients: | |  |  |  |
|  | Estimate | S.E. | t | P |
| (Intercept) | -0.04358 | 0.07161 | -0.609 | 0.543 |
| PRGS | -0.08374 | 0.07178 | -1.167 | 0.245 |
|  |  |  |  |  |
| Amygdala Neg > Pos Faces right mean | | | |  |
| Coefficients: | |  |  |  |
|  | Estimate | S.E. | t | P |
| (Intercept) | 2.752 | 1.2 | 2.293 | 0.0229 |
| PRGS | -1.579 | 1.203 | -1.313 | 0.1908 |

**Polygenic risk score vs amygdala activation in bipolar disorder sample (N=85)**

| Amygdala Neg Faces > Shapes left eigenvalue | | | |  |
| --- | --- | --- | --- | --- |
| Coefficients: | |  |  |  |
|  | Estimate | S.E. | t | P |
| (Intercept) | 0.05283 | 0.12434 | 0.425 | 0.672 |
| PGRS | -0.15933 | 0.12666 | -1.258 | 0.212 |
|  |  |  |  |  |
| Amygdala Neg Faces > Shapes left mean | | | |  |
| Coefficients: | |  |  |  |
|  | Estimate | S.E. | t | P |
| (Intercept) | 14.893 | 1.82 | 8.181 | 2.83E-12 |
| PGRS | -2.513 | 1.854 | -1.355 | 0.179 |
|  |  |  |  |  |
| Amygdala Neg Faces > Shapes right eigenvalue | | | |  |
| Coefficients: | |  |  |  |
|  | Estimate | S.E. | t | P |
| (Intercept) | 0.01886 | 0.12956 | 0.146 | 0.885 |
| PGRS | -0.08979 | 0.13198 | -0.68 | 0.498 |
|  |  |  |  |  |
| Amygdala Neg Faces > Shapes right mean | | | | |
| Coefficients: | |  |  |  |
|  | Estimate | S.E. | t | P |
| (Intercept) | 15.647 | 1.914 | 8.176 | 2.91E-12 |
| PGRS | -1.646 | 1.95 | -0.844 | 0.401 |
|  |  |  |  |  |
| Amygdala Pos Faces > Shapes left eigenvalue | | | |  |
| Coefficients: | |  |  |  |
|  | Estimate | S.E. | t | P |
| (Intercept) | -0.12931 | 0.13129 | -0.985 | 0.328 |
| PGRS | 0.08317 | 0.13374 | 0.622 | 0.536 |
|  |  |  |  |  |
| Amygdala Pos Faces > Shapes left mean | | | |  |
| Coefficients: | |  |  |  |
|  | Estimate | S.E. | t | P |
| (Intercept) | 9.679 | 1.724 | 5.615 | 2.55E-07 |
| PGRS | 1.577 | 1.756 | 0.898 | 0.372 |
|  |  |  |  |  |
| Amygdala Pos Faces > Shapes right eigenvalue | | | |  |
| Coefficients: | |  |  |  |
|  | Estimate | S.E. | t | P |
| (Intercept) | -0.15795 | 0.12142 | -1.301 | 0.197 |
| PGRS | 0.08233 | 0.12369 | 0.666 | 0.507 |
|  |  |  |  |  |
| Amygdala Pos Faces > Shapes right mean | | | |  |
| Coefficients: | |  |  |  |
|  | Estimate | S.E. | t | P |
| (Intercept) | 10.048 | 1.574 | 6.382 | 9.46E-09 |
| PGRS | 1.144 | 1.604 | 0.713 | 0.478 |
|  |  |  |  |  |
| Amygdala Faces > Shapes left eigenvalue | | | |  |
| Coefficients: | |  |  |  |
|  | Estimate | S.E. | t | P |
| (Intercept) | -0.05414 | 0.1219 | -0.444 | 0.658 |
| PGRS | -0.04655 | 0.12417 | -0.375 | 0.709 |
|  |  |  |  |  |
| Amygdala Faces > Shapes left mean | | | |  |
| Coefficients: | |  |  |  |
|  | Estimate | S.E. | t | P |
| (Intercept) | 24.572 | 2.707 | 9.076 | 4.6e-14 |
| PGRS | -0.936 | 2.758 | -0.339 | 0.735 |
|  |  |  |  |  |
| Amygdala Faces > Shapes right eigenvalue | | | |  |
| Coefficients: | |  |  |  |
|  | Estimate | S.E. | t | P |
| (Intercept) | -0.08372 | 0.13343 | -0.627 | 0.532 |
| PGRS | -0.00203 | 0.135921 | -0.015 | 0.988 |
|  |  |  |  |  |
| Amygdala Faces > Shapes right mean | | | |  |
| Coefficients: | |  |  |  |
|  | Estimate | S.E. | t | P |
| (Intercept) | 25.6951 | 2.9831 | 8.614 | 3.87E-13 |
| PGRS | -0.5023 | 3.0388 | -0.165 | 0.869 |
|  |  |  |  |  |
| Amygdala Neg > Pos Faces left eigenvalue | | | |  |
| Coefficients: | |  |  |  |
|  | Estimate | S.E. | t | P |
| (Intercept) | 0.1428 | 0.1338 | 1.067 | 0.289 |
| PGRS | -0.1704 | 0.1363 | -1.25 | 0.215 |
|  |  |  |  |  |
| Amygdala Neg > Pos Faces left mean | | | |  |
| Coefficients: | |  |  |  |
|  | Estimate | S.E. | t | P |
| (Intercept) | 5.213 | 2.289 | 2.277 | 0.0253 |
| PGRS | -4.09 | 2.332 | -1.754 | 0.0831 |
|  |  |  |  |  |
| Amygdala Neg > Pos Faces right eigenvalue | | | |  |
| Coefficients: | |  |  |  |
|  | Estimate | S.E. | t | P |
| (Intercept) | 0.1432 | 0.1088 | 1.316 | 0.192 |
| PGRS | -0.1367 | 0.1109 | -1.233 | 0.221 |
|  |  |  |  |  |
| Amygdala Neg > Pos Faces right mean | | | |  |
| Coefficients: | |  |  |  |
|  | Estimate | S.E. | t | P |
| (Intercept) | 5.598 | 1.84 | 3.043 | 0.00313 |
| PGRS | -2.789 | 1.874 | -1.488 | 0.14041 |

**Polygenic risk score vs amygdala activation in healthy controls sample (N=121)**

| Amygdala Neg Faces > Shapes left eigenvalue | | | |  |
| --- | --- | --- | --- | --- |
| Coefficients: | |  |  |  |
|  | Estimate | S.E. | t | P |
| (Intercept) | -0.03606 | 0.09313 | -0.387 | 0.699 |
| PGRS | -0.01968 | 0.09234 | -0.213 | 0.832 |
|  |  |  |  |  |
| Amygdala Neg Faces > Shapes left mean | | | |  |
| Coefficients: | |  |  |  |
|  | Estimate | S.E. | t | P |
| (Intercept) | 13.7798 | 1.32 | 10.439 | <2e-16 |
| PGRS | -0.4258 | 1.3087 | -0.325 | 0.746 |
|  |  |  |  |  |
| Amygdala Neg Faces > Shapes right eigenvalue | | | |  |
| Coefficients: | |  |  |  |
|  | Estimate | S.E. | t | P |
| (Intercept) | -0.06814 | 0.08835 | -0.771 | 0.442 |
| PGRS | -0.08906 | 0.0876 | -1.017 | 0.311 |
|  |  |  |  |  |
| Amygdala Neg Faces > Shapes right mean | | | | |
| Coefficients: | |  |  |  |
|  | Estimate | S.E. | t | P |
| (Intercept) | 14.456 | 1.302 | 11.106 | <2e-16 |
| PGRS | -1.245 | 1.291 | -0.965 | 0.337 |
|  |  |  |  |  |
| Amygdala Pos Faces > Shapes left eigenvalue | | | |  |
| Coefficients: | |  |  |  |
|  | Estimate | S.E. | t | P |
| (Intercept) | 0.07619 | 0.08703 | 0.876 | 0.383 |
| PGRS | 0.04481 | 0.08629 | 0.519 | 0.605 |
|  |  |  |  |  |
| Amygdala Pos Faces > Shapes left mean | | | |  |
| Coefficients: | |  |  |  |
|  | Estimate | S.E. | t | P |
| (Intercept) | 12.4773 | 1.2746 | 9.789 | <2e-16 |
| PGRS | 0.6822 | 1.2637 | 0.54 | 0.59 |
|  |  |  |  |  |
| Amygdala Pos Faces > Shapes right eigenvalue | | | |  |
| Coefficients: | |  |  |  |
|  | Estimate | S.E. | t | P |
| (Intercept) | 0.14149 | 0.09066 | 1.561 | 0.121 |
| PGRS | 0.04656 | 0.08989 | 0.518 | 0.605 |
|  |  |  |  |  |
| Amygdala Pos Faces > Shapes right mean | | | |  |
| Coefficients: | |  |  |  |
|  | Estimate | S.E. | t | P |
| (Intercept) | 13.4833 | 1.2292 | 10.969 | <2e-16 |
| PGRS | 0.4338 | 1.2188 | 0.356 | 0.723 |
|  |  |  |  |  |
| Amygdala Faces > Shapes left eigenvalue | | | |  |
| Coefficients: | |  |  |  |
|  | Estimate | S.E. | t | P |
| (Intercept) | 0.02269 | 0.09469 | 0.24 | 0.811 |
| PGRS | 0.01417 | 0.09389 | 0.151 | 0.88 |
|  |  |  |  |  |
| Amygdala Faces > Shapes left mean | | | |  |
| Coefficients: | |  |  |  |
|  | Estimate | S.E. | t | P |
| (Intercept) | 26.257 | 2.1113 | 12.436 | <2e-16 |
| PGRS | 0.2565 | 2.0933 | 0.123 | 0.903 |
|  |  |  |  |  |
| Amygdala Faces > Shapes right eigenvalue | | | |  |
| Coefficients: | |  |  |  |
|  | Estimate | S.E. | t | P |
| (Intercept) | 0.04132 | 0.08427 | 0.49 | 0.625 |
| PGRS | -0.02986 | 0.08355 | -0.357 | 0.721 |
|  |  |  |  |  |
| Amygdala Faces > Shapes right mean | | | |  |
| Coefficients: | |  |  |  |
|  | Estimate | S.E. | t | P |
| (Intercept) | 27.9398 | 1.9214 | 14.541 | <2e-16 |
| PGRS | -0.8117 | 1.9051 | -0.426 | 0.671 |
|  |  |  |  |  |
| Amygdala Neg > Pos Faces left eigenvalue | | | |  |
| Coefficients: | |  |  |  |
|  | Estimate | S.E. | t | P |
| (Intercept) | -0.08937 | 0.0841 | -1.063 | 0.29 |
| PGRS | -0.04426 | 0.08338 | -0.531 | 0.597 |
|  |  |  |  |  |
| Amygdala Neg > Pos Faces left mean | | | |  |
| Coefficients: | |  |  |  |
|  | Estimate | S.E. | t | P |
| (Intercept) | 1.303 | 1.509 | 0.863 | 0.39 |
| PGRS | -1.108 | 1.496 | -0.741 | 0.46 |
|  |  |  |  |  |
| Amygdala Neg > Pos Faces right eigenvalue | | | |  |
| Coefficients: | |  |  |  |
|  | Estimate | S.E. | t | P |
| (Intercept) | -0.16945 | 0.09853 | -1.72 | 0.0881 |
| PGRS | -0.10975 | 0.0977 | -1.123 | 0.2635 |
|  |  |  |  |  |
| Amygdala Neg > Pos Faces right mean | | | |  |
| Coefficients: | |  |  |  |
|  | Estimate | S.E. | t | P |
| (Intercept) | 0.9731 | 1.649 | 0.59 | 0.556 |
| PGRS | -1.6793 | 1.6349 | -1.027 | 0.306 |

**Mean amygdala activation across diagnostic groups**

| Means of amygdala Neg Faces > Shapes left eigenvalue VS diagnosis | | |
| --- | --- | --- |
|  | Mean | S.D. |
| BD1 | -0.05987 | 1.024366 |
| BD2 | -0.01921 | 0.954869 |
| BDNOS | 0.652763 | 2.153187 |
| HC | -0.03214 | 1.000295 |
|  |  |  |
| Means of amygdala Neg Faces > Shapes left eigenvalue VS case-control status | | |
|  | Mean | S.D. |
| Controls | -0.03214 | 1.000295 |
| Cases | 0.007656 | 1.101319 |
|  |  |  |
| Means of amygdala Neg Faces > Shapes left mean VS diagnosis | | |
|  | Mean | S.D. |
| BD1 | 13.95893 | 14.71247 |
| BD2 | 12.88048 | 13.82982 |
| BDNOS | 23.56039 | 33.24219 |
| HC | 13.86459 | 14.18068 |
|  |  |  |
| Means of amygdala Neg Faces > Shapes left mean VS case-control status | | |
|  | Mean | S.D. |
| Controls | 13.86459 | 14.18068 |
| Cases | 14.17992 | 16.1472 |
|  |  |  |
| Means of amygdala Neg Faces > Shapes right eigenvalue VS diagnosis | | |
|  | Mean | S.D. |
| BD1 | -0.09149 | 0.888339 |
| BD2 | -0.04052 | 1.10691 |
| BDNOS | 0.805199 | 2.400542 |
| HC | -0.0504 | 0.952855 |
|  |  |  |
| Means of amygdala Neg Faces > Shapes right eigenvalue VS case-control status | | |
|  | Mean | S.D. |
| Controls | -0.0504 | 0.952855 |
| Cases | -0.0066 | 1.139894 |
|  |  |  |
| Means of amygdala Neg Faces > Shapes right mean VS diagnosis | | |
|  | Mean | S.D. |
| BD1 | 14.42254 | 12.82996 |
| BD2 | 14.21324 | 17.18872 |
| BDNOS | 26.40941 | 34.11232 |
| HC | 14.70454 | 14.03285 |
|  |  |  |
| Means of amygdala Neg Faces > Shapes right mean VS case-control status | | |
|  | Mean | S.D. |
| Controls | 14.70454 | 14.03285 |
| Cases | 15.18003 | 16.86343 |
|  |  |  |
| Means of amygdala Pos Faces > Shapes left eigenvalue VS diagnosis | | |
|  | Mean | S.D. |
| BD1 | -0.09105 | 0.957494 |
| BD2 | -0.16911 | 1.352159 |
| BDNOS | 0.169355 | 1.332313 |
| HC | 0.067268 | 0.935583 |
|  |  |  |
| Means of amygdala Pos Faces > Shapes left eigenvalue VS case-control status | | |
|  | Mean | S.D. |
| Controls | 0.067268 | 0.935583 |
| Cases | -0.10573 | 1.154599 |
|  |  |  |
| Means of amygdala Pos Faces > Shapes left mean VS diagnosis | | |
|  | Mean | S.D. |
| BD1 | 10.45852 | 13.00776 |
| BD2 | 9.105148 | 17.25724 |
| BDNOS | 13.87584 | 18.69051 |
| HC | 12.34137 | 13.70346 |
|  |  |  |
| Means of amygdala Pos Faces > Shapes left mean VS case-control status | | |
|  | Mean | S.D. |
| Controls | 12.34137 | 13.70346 |
| Cases | 10.12655 | 15.19838 |
|  |  |  |
| Means of amygdala Pos Faces > Shapes right eigenvalue VS diagnosis | | |
|  | Mean | S.D. |
| BD1 | -0.12404 | 1.069785 |
| BD2 | -0.18325 | 1.093242 |
| BDNOS | 0.081515 | 1.055971 |
| HC | 0.132213 | 0.974655 |
|  |  |  |
| Means of amygdala Pos Faces > Shapes right eigenvalue VS case-control status | | |
|  | Mean | S.D. |
| Controls | 0.132213 | 0.974655 |
| Cases | -0.13461 | 1.068172 |
|  |  |  |
| Means of amygdala Pos Faces > Shapes right mean VS diagnosis | | |
|  | Mean | S.D. |
| BD1 | 11.17767 | 13.89685 |
| BD2 | 9.077858 | 14.04703 |
| BDNOS | 12.37153 | 14.12418 |
| HC | 13.39694 | 13.20682 |
|  |  |  |
| Means of amygdala Pos Faces > Shapes right mean VS case-control status | | |
|  | Mean | S.D. |
| Controls | 13.39694 | 13.20682 |
| Cases | 10.37261 | 13.85596 |
|  |  |  |
| Means of amygdala Faces > Shapes left eigenvalue VS diagnosis | | |
|  | Mean | S.D. |
| BD1 | -0.09567 | 0.915009 |
| BD2 | -0.13169 | 1.073676 |
| BDNOS | 0.521849 | 1.921953 |
| HC | 0.01987 | 1.016954 |
|  |  |  |
| Means of amygdala Faces > Shapes left eigenvalue VS case-control status | | |
|  | Mean | S.D. |
| Controls | 0.01987 | 1.016954 |
| Cases | -0.06734 | 1.070421 |
|  |  |  |
| Means of amygdala Faces > Shapes left mean VS diagnosis | | |
|  | Mean | S.D. |
| BD1 | 24.41744 | 20.73186 |
| BD2 | 21.98563 | 23.06267 |
| BDNOS | 37.43623 | 43.74046 |
| HC | 26.20596 | 22.67345 |
|  |  |  |
| Means of amygdala Faces > Shapes left mean VS case-control status | | |
|  | Mean | S.D. |
| Controls | 26.20596 | 22.67345 |
| Cases | 24.30647 | 23.77047 |
|  |  |  |
| Means of amygdala Faces > Shapes right eigenvalue VS diagnosis | | |
|  | Mean | S.D. |
| BD1 | -0.12962 | 1.021499 |
| BD2 | -0.13796 | 1.176282 |
| BDNOS | 0.562532 | 2.008435 |
| HC | 0.047267 | 0.905387 |
|  |  |  |
| Means of amygdala Faces > Shapes right eigenvalue VS case-control status | | |
|  | Mean | S.D. |
| Controls | 0.047267 | 0.905387 |
| Cases | -0.08429 | 1.170694 |
|  |  |  |
| Means of amygdala Faces > Shapes right mean VS diagnosis | | |
|  | Mean | S.D. |
| BD1 | 25.6002 | 22.29337 |
| BD2 | 23.2911 | 27.09463 |
| BDNOS | 38.78093 | 44.17905 |
| HC | 28.10148 | 20.64849 |
|  |  |  |
| Means of amygdala Faces > Shapes right mean VS case-control status | | |
|  | Mean | S.D. |
| Controls | 28.10148 | 20.64849 |
| Cases | 25.55263 | 26.17728 |
|  |  |  |
| Means of amygdala Neg > Pos Faces left eigenvalue VS diagnosis | | |
|  | Mean | S.D. |
| BD1 | 0.010437 | 1.088771 |
| BD2 | 0.150854 | 1.272898 |
| BDNOS | 0.358355 | 1.458532 |
| HC | -0.08055 | 0.90415 |
|  |  |  |
| Means of amygdala Neg > Pos Faces left eigenvalue VS case-control status | | |
|  | Mean | S.D. |
| Controls | -0.08055 | 0.90415 |
| Cases | 0.094467 | 1.185074 |
|  |  |  |
| Means of amygdala Neg > Pos Faces left mean VS diagnosis | | |
|  | Mean | S.D. |
| BD1 | 3.500402 | 18.47993 |
| BD2 | 3.77533 | 21.12499 |
| BDNOS | 9.684555 | 31.55202 |
| HC | 1.523213 | 16.23785 |
|  |  |  |
| Means of amygdala Neg > Pos Faces left mean VS case-control status | | |
|  | Mean | S.D. |
| Controls | 1.523213 | 16.23785 |
| Cases | 4.053371 | 20.45508 |
|  |  |  |
| Means of amygdala Neg > Pos Faces right eigenvalue VS diagnosis | | |
|  | Mean | S.D. |
| BD1 | 0.003665 | 0.895122 |
| BD2 | 0.137234 | 0.901167 |
| BDNOS | 0.630334 | 1.659479 |
| HC | -0.14758 | 1.063689 |
|  |  |  |
| Means of amygdala Neg > Pos Faces right eigenvalue VS case-control status | | |
|  | Mean | S.D. |
| Controls | -0.14758 | 1.063689 |
| Cases | 0.104471 | 0.9635 |
|  |  |  |
| Means of amygdala Neg > Pos Faces > Shapes right mean VS diagnosis | | |
|  | Mean | S.D. |
| BD1 | 3.244869 | 14.78061 |
| BD2 | 5.135385 | 15.85634 |
| BDNOS | 14.03788 | 27.82977 |
| HC | 1.307581 | 17.78541 |
|  |  |  |
| Means of amygdala Neg > Pos Faces right mean VS case-control status | | |
|  | Mean | S.D. |
| Controls | 1.307581 | 17.78541 |
| Cases | 4.807418 | 16.35469 |

Abbreviations: BD, bipolar disorder; NOS, not otherwise specified; HC, healthy controls; Neg, Negative; Pos, Positive; PGRS, polygenic risk score.
